# Gentamicin loading of calcium phosphate implants: implications for cranioplasty

**DOI:** 10.1007/s00701-019-03895-4

**Published:** 2019-04-30

**Authors:** Jimmy Sundblom, Sara Gallinetti, Ulrik Birgersson, Håkan Engqvist, Lars Kihlström

**Affiliations:** 1 Department of Neuroscience, Neurosurgery, Uppsala University Hospital, Akademiska sjukhuset, 751 85 Uppsala, Sweden; 2 Department of Engineering Sciences, Applied Materials Science Division, Uppsala University, Uppsala, Sweden; 3 Department of Clinical Science, Intervention and Technology, Division of Imaging and Technology, Karolinska Institutet, Huddinge, Sweden; 4 Department of Neurosurgery, Clinical Neurosciences, Karolinska University Hospital and Karolinska Institutet, Stockholm, Sweden

**Keywords:** Gentamicin, Calcium phosphate, Titanium, PEEK, Staphylococcus, Uptake-release

## Abstract

**Background:**

Surgical site infections (SSI) are a significant risk in cranioplasty, with reported rates of around 8–9%. The most common bacteria associated with these nosocomial infections are of the Staphylococcus species, which have the ability to form biofilm. The possibility to deliver antibiotics, such as gentamicin, locally rather than systemically could potentially lower the early postoperative SSI. Various antibiotic dosages are being applied clinically, without any true consensus on the effectiveness.

**Methods:**

Drug release from calcium phosphate (CaP), polyetheretherketone (PEEK), and titanium (Ti) samples was evaluated. Microbiological studies with *Staphylococcus aureus* (SA) and *Staphylococcus epidermidis* (SE) including strains from clinical infection were used to establish clinically relevant concentrations.

**Results:**

The CaP samples were able to retain and release gentamicin overtime, whereas the Ti and PEEK samples did not show any drug uptake or release. A gentamicin loading concentration of 400 μg/ml was shown to be effective in in vitro microbiological studies with both SA and SE.

**Conclusions:**

Out of the three materials studied, only CaP could be loaded with gentamicin. An initial loading concentration of 400 μg/ml appears to establish an effective gentamicin concentration, possibly translating into a clinical benefit in cranioplasty.

## Introduction

Surgical site infections (SSI) in cranioplasty pose a significant risk with reported rates of around 8–9% [1–7]. The most common bacteria associated with nosocomial infections are of the Staphylococcus species. Out of these, the most predominant are *Staphylococcus epidermidis* (SE) and *Staphylococcus aureus* (SA) [4, 8, 9]. They are generally carried asymptomatically on the skin, especially in moist skin regions [4, 8–10]. Both SE [11] and SA [12] bacteria strains are able to form biofilms, which was recently associated with 65% of all the microbial bone infections treated by clinicians in the developed world [13]. Host immune responses against persistent biofilm infections are largely ineffective and can lead to chronic infection, which is associated with difficult post-op treatments [12]. Antibiotic prophylaxis is usually administered before cranioplasty, even though its use is still debated, due to the risk of creating resistant strains if administrated inadequately [14].

Antibiotics can be delivered both systemically and/or locally. The advantages of local administration over systemic are lower cost, lower risk of toxicity, and significantly higher concentrations of antibiotics at the desired site [15]. Loading a device with antibiotics could allow for a lower systemic concentration while obtaining the same effect locally, reducing the drawbacks of systemic delivery, including development of resistant strains. For these reasons, local delivery of drugs has been applied into different applications, such as in orthopedics or dentistry [16, 17]. In order to obtain an effective treatment as well as limiting the drugs cytotoxic effects, a critical concentration between 8 to 16 μg/ml and 100 μg/ml seems to be necessary [11, 18–23].

Calcium phosphate (CaP) cements have been loaded with vancomycin and gentamicin in order to decrease the number of bone infections, especially in orthopedics [16]. The antibiotics have been added to the cement either in liquid form [24, 25] or as a solid phase [26, 27]. Antibiotic immersion of titanium (Ti) implants such as pedicle screws used in spine surgery has been tested and showed decreased rates of SSI [28]. Gentamicin is one of the most commonly used antibiotics in cranioplasty and the focus of this paper. While efficient against infections, gentamicin has been shown to negatively influence cell proliferation [19, 20, 22] making the tuning of the concentration vital.

The aim of the study was to evaluate the drug uptake and release for the three most widespread commercially available materials used for patient-specific custom-made implants for cranioplasty, namely CaP, polyetheretherketone (PEEK), and Ti, and to test the bactericidal effect against strains of SA and SE.

## Material and methods

### In vitro studied materials

Hexagonal CaP cement tiles (OssDsign AB) composed of monetite, calcium pyrophosphate and beta tricalcium phosphate [29], and medical grade PEEK (ESSADE AB) and Ti medical grade 5 (Livallco stål AB) discs with diameter Φ = 10 mm and height *h* = 6 mm were purchased. All samples were steam sterilized.

The porosity was measured using the Archimedes method in distilled water [30]. Specific surface area (SSA) of CaP was determined by nitrogen adsorption at 77 K according to the Brunnauer–Emmet–Teller method (BET) [31] in an ASAP 2020 (Micromeritics).

### Gentamicin in vitro uptake-release

Gentamicin (Sanofi AB) was mixed with Ringer’s solution (Baxter) at concentrations of 200 μg/ml and 400 μg/ml. The drug concentrations were tested for CaP, PEEK, and Ti. Samples were loaded by soaking in the solution at room temperature for 15 min (time to reach saturation). Control samples were soaked in Ringer’s only.

The samples were transferred after loading into 4 ml PBS solution (Sigma Aldrich) at 37 °C for the release with slow orbital media agitation. Two hundred microliter samples were withdrawn after 0.5 h, 1 h, 2 h, 4 h, 6 h, 24 h, and 48 h and replaced with fresh media at 37 °C. The samples were stored at 4 °C protected from light prior to analysis. Gentamicin amount was quantified using a colorimetric method using spectrophotometry based on o–phthaldialdehyde reaction with gentamicin amino groups (Sampath [32] with modifications by Zhang [33]) considering the volume sampling.

### Agar diffusion test

Gentamicin solutions in Ringer’s (Baxter) were prepared at concentrations of 200 μg/ml and 400 μg/ml. The solutions were filtered with a 0.2-μm sterile filter (Thermo Fisher Scientific). Control samples were loaded with Ringer solution only. Three different bacteria strains were tested: Pharmacopeia US and EU standard for *Staphylococcus aureus* (SA, ATCC 6538) and *Staphylococcus epidermidis* (SE, ATCC 14990), and a SA strain derived from a clinical case of postoperative infection including empyema after revision cranioplasty with custom made calcium phosphate implant (OssDsign AB). Subsequent revision with an identical implant was uneventful. Gentamicin soaking of the implant was performed at both surgical procedures, but postoperative images after initial surgery revealed significant dead space beneath the cranioplasty.

Bacteria were seeded at a concentration of 10^6^ CFU on agar (ISO-SENSITEST AGAR, Oxoid) plates. The plates were incubated at 32.5 ± 2.5 °C for 20 h. The inhibition zones were calculated and compared with the performance standards for antimicrobial susceptibility testing according to the Clinical and Laboratory Standard Institute (2013).

### Statistics

Statistical differences were determined using one-way ANOVA with Tukey’s post-hoc test (95%) using Minitab 18 software (Minitab Inc., State College, PA). Statistical significance was indicated when *p* < 0.05.

## Results

### Material characterization

Total porosity of CaP cement was 42.3% ± 1.4%, PEEK was 0.5% ± 0.5%, while the one of titanium (Ti) was 0.7% ± 0.3%. CaP are intrinsically porous, which allows aqueous solution uptake. This porous microstructure of the CaP and its interlocking crystals gives a surface area of approximately 4 m^2^/g.

### Antibiotic release

**Fig. 1:**
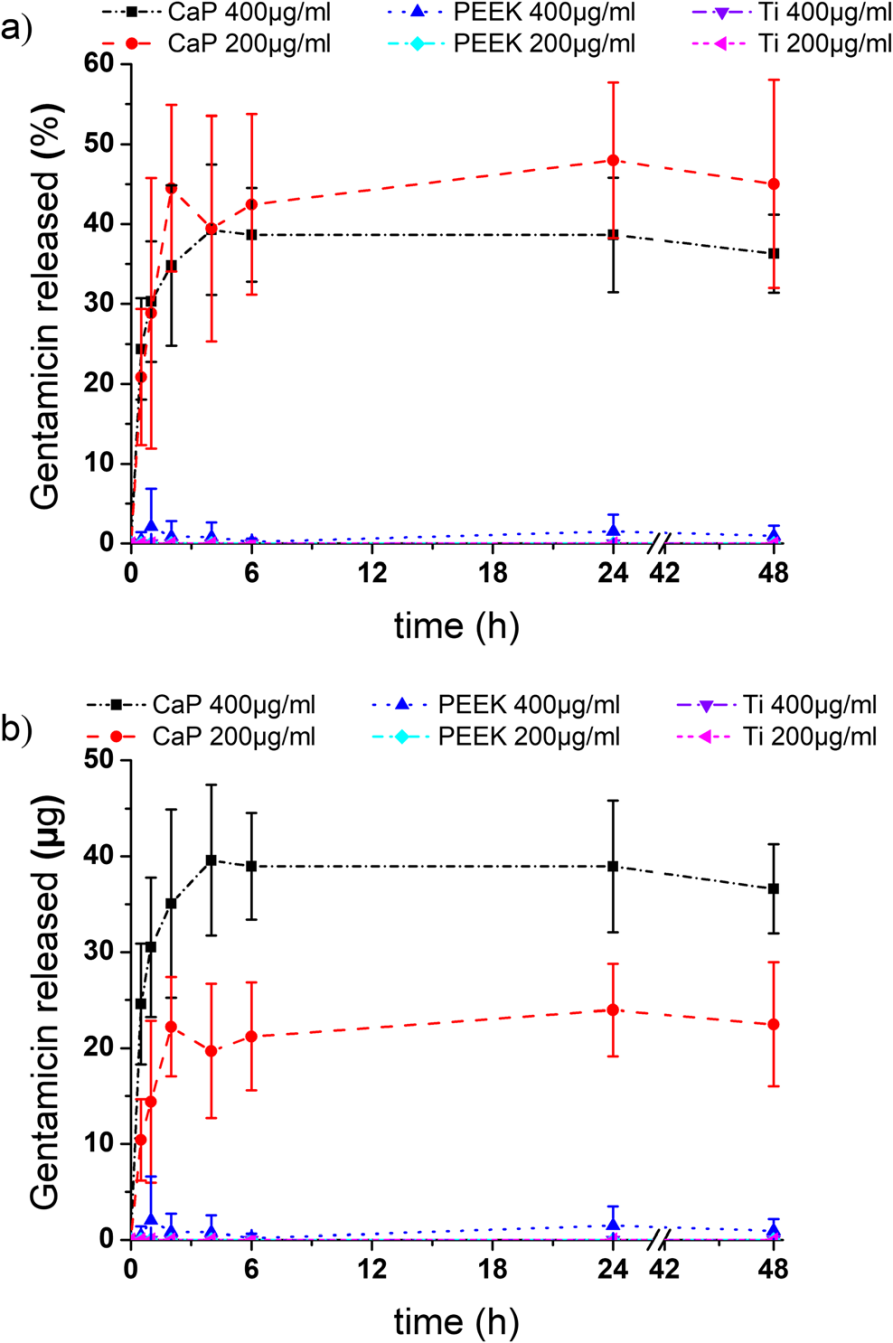
Release from CaP tiles (CaP 200 μg/ml), PEEK (PEEK 200 μg/ml), and Ti (Ti 200 μg/ml) loaded with a gentamicin solution of 200 μg/ml; and CaP (CaP 400 μg/ml), PEEK (PEEK 400 μg/ml), and Ti (Ti 400 μg/ml), respectively, loaded with a gentamicin solution of 400 μg/ml displayed as **a** percentage released and **b** amount of drug released

The release profile of the antibiotic for CaP was characterized by a burst release within the first 24 h of 48 ± 10% for 200 μg/ml and 39 ± 7% for 400 μg/ml, followed by a gradual stabilization. Within the timeframe, no measurable drug degradation occurred. Zero to minimal release was measured for Ti and PEEK (Fig. 1).

Agar diffusion tests for CaP showed that there was a slight difference in the inhibition zone at low concentrations but not significant (*p* > 0.05), as expected, the higher the concentration, the wider the inhibition zone. Here, it is noteworthy that the SA strain derived from the patient was less sensitive to the drug than ATCC 6538 but still in the range of sensitivity. For the SE strain, both gentamicin concentrations used for loading the CaP were effective.

## Discussion

In comparison to solid implants, a major advantage of a porous material is its ability to absorb liquids, allowing them to be used as drug carriers. By pre-loading a cranial implant, local delivery of antibiotics can be achieved which is more effective and less harmful for the body [16].

In this study, the CaP material characteristics result in a large surface area which facilitates a reasonably quick drug uptake (saturation is reached after 15 min soaking in aqueous solution), while in the case of both PEEK and titanium gentamicin loading remains difficult to achieve. Whether this immediately translates to a clinical advantage is possible but has not been addressed in this study.

The release profile of gentamicin for CaP was characterized by a burst release within the first 24 h followed by a gradual stabilization. Noteworthy, the CaP retained a high amount of gentamicin, likely due to a strong affinity of the drug to the sample, which is similar to the observations made by Canal et al. [34] for a different drug with a similar CaP composition. Therefore, the loading and release profile for different antibiotics and concentrations has to be evaluated in vitro to ensure that clinically relevant concentrations can be obtained in vivo. Even though it did not come as a surprise that there was no gentamicin uptake or release for both titanium and PEEK, however, they were still evaluated in this study as it remains common practice to pre-treat different metal [28] and plastic implants in the OR with antibiotics even if its efficacy is mostly unknown.

An initial concentration of 400 μg/ml for the CaP implant would not be harmful for the cells as this would translate into a concentration of approximately 80 μg/ml after 6 h given that in the first 6 h around 40% of the drug is released and diluted by half when taking the space between the skin and dura and inter-tile spaces into consideration. In the presence of additional dead space, the gentamicin concentration would be further diluted and thus impacting the drug’s efficacy. In clinical use, many other factors could possibly influence the drug concentration, such as the use of wound drain, drug absorption by surrounding vascularized tissue, and use of suction during wound closure, making the systematic use of this feature of CaP implants contingent on further in vivo studies and randomized trials.

## Conclusions

Out of the three clinically used, and here studied, materials only CaP could be loaded with gentamicin. This observed feature may create a substantial clinical opportunity in cranioplasty with potential of limiting the number of infections. An initial loading concentration of 400 μg/ml can potentially reduce surgical site infections, establishing an effective localized gentamicin concentration sustained over time. To truly appraise the potential clinical benefits of gentamicin loading, a randomized trial is needed.

## Abbreviations

ATCC: American Type Culture Collection
BET: Brunnauer–Emmet–Teller method
CaP: Calcium phosphate
CFU: Colony-forming unit
EU: European Union
PEEK: Polyetheretherketone
SA: *Staphylococcus aureus*
SE: *Staphylococcus epidermidis*
SSA: Specific surface area
SSI: Surgical site infections
Ti: Titanium
US: United States of America
